# Application of Prospect Theory in Obstetrics by Evaluating Mode of Delivery and Outcomes in Neonates Born Small or Appropriate for Gestational Age

**DOI:** 10.1001/jamanetworkopen.2022.2177

**Published:** 2022-03-15

**Authors:** Jennia Michaeli, Ofir Michaeli, Ariel Rozitzky, Sorina Grisaru-Granovsky, Naomi Feldman, Naama Srebnik

**Affiliations:** 1 Department of Obstetrics and Gynecology, Shaare Zedek Medical Center Affiliated with the Hebrew University Hadassah School of Medicine, Jerusalem, Israel; 2 Mount Sinai Fertility, Department of Obstetrics and Gynaecology, University of Toronto, Toronto, Ontario, Canada; 3 Department of Economics, Hebrew University of Jerusalem, Jerusalem, Israel

## Abstract

**Importance:**

Antenatal diagnosis of fetal weight is challenging, and the detection rate of fetal growth restriction (FGR) is low. Neonates with FGR are known to have an increased rate of obstetric intervention during labor, but the association of antenatal fetal weight estimation with mode of delivery and neonatal outcomes among neonates who are small and appropriate for gestational age (SGA and AGA) has not been reported.

**Objective:**

To evaluate the association of antenatal fetal weight estimation with mode of delivery and neonatal outcomes among neonates who are SGA and AGA, applying psychological concepts of cognitive bias and prospect theory to a model of clinical behavior.

**Design, Setting, and Participants:**

This cohort study was conducted between 2019 and 2020 using data from 2006 to 2018 at a tertiary care center in Jerusalem, Israel. Participants were 100 198 term singleton neonates without anomalies who were categorized into 4 groups according to the presence of an antenatal suspicion of FGR and final birth weight. Neonates with false positives (FPs; ie, group 1-FP: those with suspected FGR who were AGA) and neonates with true positives (TPs; ie, group 2-TP: those with suspected FGR who were SGA) were compared with neonates with AGA antenatal fetal weight estimation, including neonates with false negatives (FNs; ie, group 3-FN: those not suspected to have FGR who were SGA) and neonates with true negatives (TNs; ie, group 4-TN: those not suspected to have FGR who were AGA). Data were analyzed from July 2019 to July 2020.

**Exposures:**

Fetal weight estimation was performed according to sonographic and clinical evaluation at admission to labor, with FGR defined as a birth weight less than the 10th percentile for gestational age. Sonographic fetal weight estimation was performed according to Hadlock formula. Clinical weight estimation was performed by trained obstetricians.

**Main Outcomes and Measures:**

The primary outcomes were obstetric intervention and mode of delivery; the secondary outcomes were neonatal Apgar score (with low Apgar score defined as <7) and neonatal intensive care unit (NICU) admission rates.

**Results:**

Among 100 198 neonates eligible for the study (50941 [50.8%] male neonates), there were 5671 neonates in group 1-FP, 3040 neonates in group 2-TP, 8508 neonates in group 3-FN, and 82 979 neonates in group 4-TN. Mean (SD) maternal age was 28.6 (5.7) years. Among 8711 neonates with suspected FGR, 34.9% were below the 10th percentile at birth, while 65.1% were AGA. Neonates with suspected FGR had a significantly increased rate of induction of labor (group 1-FP: 649 neonates [11.4%] and group 2-TP: 969 neonates [31.9%]) compared with neonates in group 3-FN (1055 neonates [12.4%]) and group 4-TN (7136 neonates [8.6%]) (*P* < .001) and a significantly increased rate of cesarean delivery (group 1-FP: 915 neonates [16.1%] and group 2-TP: 556 neonates [18.3%] vs group 3-FN: 1106 neonates [13.0%] and group 4-TN: 6588 neonates [7.9%]; *P* < .001). Increased NICU admission was found for neonates who were SGA compared with neonates who were AGA (group 2-TP: 182 neonates [6.0%] and group 3-FN: 328 neonates [3.9%] vs group 1-FP: 51 neonates [0.9%] and group 4-TN: 704 neonates [0.8%]; *P* <.001), as was increased rate of low Apgar score (eg, at 1 minute: group 2-TP: 149 neonates [4.9%] and group 3-FN: 384 neonates [4.5%] vs group 1-FP: 124 neonates [2.2%] and group 4-TN: 1595 neonates [1.9%]; *P* < .001). In a multivariable model comparing group 1-FP, group 2-TP, and group 3-FN with group 4-TN, suspicion of FGR was independently associated with increased risk of caesarean delivery among neonates in group 1-FP (odds ratio, 1.72; 95% CI, 1.56-1.88; *P* < .001).

**Conclusions and Relevance:**

This study found that antenatal diagnosis of FGR was independently associated with an increase in risk of caesarean delivery by 70% in neonates who were AGA without improvement in neonatal outcomes. These findings suggest that such outcomes may be explained by application of prospect theory and may be associated with cognitive bias in clinical decision-making.

## Key Points

**Question:**

Is antenatal fetal weight estimation associated with mode of delivery and neonatal outcomes of neonates who are small and appropriate for gestational age (SGA and AGA)?

**Findings:**

In this cohort study of 100 198 neonates, suspicion of fetal growth restriction (FGR) in neonates who were AGA was associated with cognitive bias in clinicians and with an increase in risk for cesarean delivery by more than 70%. Regardless of antenatal diagnosis of FGR, neonates who were SGA had increased rates of low Apgar scores and increased neonatal intensive care unit admission.

**Meaning:**

These findings suggest that decision-making under risk circumstances among obstetricians may be associated with increased interventions but not improved neonatal outcomes and that such outcomes may be explained by application of prospect theory.

## Teaser

This cohort study investigates the association of antenatal fetal growth restriction with mode of delivery and outcomes among neonates who are small or appropriate for gestational age.

## Introduction

In accordance with the international definitions of fetal and neonatal weight, the term *fetal growth restriction* (FGR) is used to describe fetuses with an estimated fetal weight that is less than the 10th percentile for gestational age. The term *small for gestational age* (SGA) represents newborns whose birth weight is less than the 10th percentile for gestational age.^1^ Birth weight from the 10th to 90th percentile is defined appropriate for gestational age (AGA).

Neonates who are SGA have increased rates of perinatal and long-term complications. Timely recognition of fetal compromise and delivery may be warranted in order to prevent intrauterine death or long-term disability.^2,3^

The antenatal diagnosis of fetal weight is challenging and is based on the combination of sonographic and clinical estimations.^4,5^ The detection rate of FGR is reported to be below 40%.^6,7,8,9^

Various international guidelines do not indicate cesarean delivery (CD) for fetuses with FGR; however, more obstetric interventions during labor and delivery are reported among these fetuses,^10,11^ with approximately 30% of fetuses with FGR estimated to be delivered via CD.^12,13^ In addition to the obvious detrimental associations of CD and operative vaginal deliveries with maternal morbidity, the neonatal benefit of CD in neonates with FGR remains unclear, and the optimal mode of delivery of neonates who are SGA remains to be determined.^14,15^

Given that definite fetal weight is unknown prior to delivery, obstetric clinical decision-making is primarily based on antenatal assessment of fetal and maternal status. This situation can be viewed as an extension of prospect theory: making decisions under risk with uncertain outcomes while subjected to major biases, such as confirmation bias. We sought to investigate cognitive bias associated with fetal weight estimation and other maternal characteristics by evaluating mode of delivery and neonatal outcomes of neonates who were SGA and AGA.

## Methods

This retrospective cohort study was conducted at a single tertiary center, Shaare Zedek Medical Center, between January 1, 2006, and December 31, 2018. Ethical committee approval was obtained from the Shaare Zedek Medical Center institutional review board. Informed consent was waived by the ethical committee given that the study exclusively used deidentified retrospective data. This study follows the Strengthening the Reporting of Observational Studies in Epidemiology (STROBE) reporting guideline for cohort studies.

Singleton, term neonates (ie, >37 weeks and 0 days of gestation) without anomalies were categorized according to the presence of antenatal suspicion of FGR and actual birth weight. FGR was defined as fetal weight estimation less than the 10th percentile and was based on nationally accepted population growth curves.^16^ Neonates with fetal weight estimation and birth weight above the 75th percentile were excluded. Data regarding fetal weight estimation, according to sonographic and clinical evaluation at admission to labor and birth weight, were retrieved from electronic health records. Sonographic fetal weight estimation was performed according to the Hadlock formula.^17^ Clinical weight estimation was performed by trained obstetricians.

Neonates with FGR were categorized into 2 groups according to birth weight: neonates with false positives (FPs; ie, group 1-FP: those with suspected FGR who were AGA) and neonates with true positives (TPs; ie, group 2-TP: those with suspected FGR who were SGA). These neonates were compared with neonates with an AGA fetal weight estimation: neonates with false negatives (FNs; ie, group 3-FN: those not suspected to have FGR who were SGA) and neonates with true negatives (TNs; ie, group 4-TN: those not suspected to have FGR who were AGA [10th -75th percentiles]) (Figure). The primary outcome was obstetric intervention and mode of delivery; the secondary outcomes were neonatal Apgar score (with low Apgar scored defined as <7) and neonatal intensive care unit (NICU) admission rates.

**Figure.**
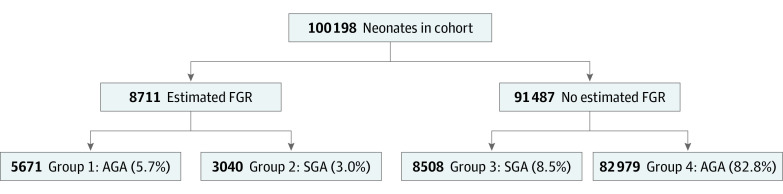
Study Design and Study Group Descriptions AGA indicates appropriate for gestational age (ie, 10th percentile < birth weight < 75th percentile population growth curves); FGR, fetal growth restriction (ie, <10th percentile population growth curves); SGA, small for gestational age (ie, birth weight <10th percentile population growth curves).

### Statistical Analysis

For categorical variables, statistics are provided as numbers and percentages. For continuous variables, statistics are described by mean values with SDs or median values with IQRs.

Comparisons between groups were performed using Pearson χ^2^ test for categorical variables. The association of categorical variables with continuous measurements was tested by 1-way analysis of variance and Kruskal-Wallis tests.

Multivariable logistic regression models were fitted to estimate the independent association of antenatal weight estimation with rates of CDs and instrumental deliveries (IDs). Models were adjusted for the following maternal and neonatal covariates: maternal age, nulliparity, prior CD, prior abortion, maternal hypertensive disorder, use of assisted reproductive technology (ART), any background maternal clinical condition, and neonatal birth weight. Models were conducted in 2 blocks. Block 1 included group 1-FP, group 2-TP, and group 3-FN, with group 4-TN as the reference group. The 3 variables were forced into the model using the enter method. Block 2 included further adjustment for maternal and gestational characteristics and was performed using the backward stepwise (or logistic regression) method. Odds ratios (ORs) and 95% CIs were reported.

All tests were 2-sided. A *P* value < .05 was considered statistically significant. Analyses were carried out using SPSS Statistics for Windows version 25.0 (IBM). Data were analyzed from July 2019 to July 2020.

## Results

### Description of Study Population

We identified 100 198 neonates (50941 [50.8%] male neonates) eligible for the study. This included 5671 neonates in group 1-FP, 3040 neonates in group 2-TP, 8508 neonates in group 3-FN, and 82 979 neonates in group 4-TN (Figure).

### Maternal, Labor, and Delivery Characteristics

Mean (SD) maternal age was 28.6 (5.7) years. Study groups were different in several maternal parameters. Among mothers of neonates in group 1-FP and group 2-TP, compared with group 3-FN and group 4-TN, there were significantly increased rates of nulliparity (1429 mothers [25.2%] and 1266 mothers [41.6%] vs 3275 mothers [38.5%] and 19 667 mothers [23.7%]; *P* < .001), hypertensive disorder (142 mothers [2.5%] and 155 mothers [5.1%] vs 285 mothers [3.3%] and 1420 mothers [1.7%]; *P* < .001), ART (208 mothers [3.7%] and 154 mothers [5.1%] vs 342 mothers [4.0%] and 2281 mothers [2.7%]; *P* < .001), and prior CD (935 mothers [16.5%] and 339 mothers [11.2%] vs 899 mothers [10.6%] and 8927 mothers [10.8%]; *P* < .001).

Neonates with suspected FGR (ie, those in study group 1-FP and group 2-TP) were different in several parameters from neonates without suspected FGR (ie, those in group 3-FN and group 4-TN) regardless of birth weight. Those in group 1-FP and group 2-TP, compared with group 3-FN and group 4-TN, had a significantly increased rate of induction of labor (649 neonates [11.4%] and 969 neonates [31.9%] vs 1055 neonates [12.4%] and 7136 neonates [8.6%]; *P* <.001), elective primary CD (679 neonates [12.0%] and 290 neonates [9.5%] vs 498 neonates [5.9%] and 3782 neonates [4.6%]; *P* <.001), and overall CD (915 neonates [16.1%] and 556 neonates [18.3%] vs 1106 neonates [13.0%] and 6588 neonates [7.9%]; *P* <.001). Neonates who were SGA (ie, those in group 2-TP and group 3-FN) had significantly increased rates of ID compared with neonates who were AGA (ie, those in group 1-FP and group 4-TN) (241 deliveries [7.9%] and 728 deliveries [8.6%] vs 248 deliveries [4.4%] and 4546 deliveries [5.5%]; *P* < .001), third-stage placental complications (ie, retained placenta or placental fragments; 104 deliveries [3.4] and 295 deliveries [3.5%] vs 148 deliveries [2.6%] and 2358 deliveries [2.8%]; *P* <.001), maternal hemoglobin drop greater than 3 g/dL (to convert to grams per liter, multiply by 10.0; 189 deliveries [6.2] and 595 deliveries [7.0%] vs 305 deliveries [5.4%] and 4906 deliveries [5.9%]; *P* < .001), and maternal need for blood transfusions (36 deliveries [1.2%] and 93 deliveries [1.1%] vs 50 deliveries [0.9%] and 701 deliveries [0.8%]; *P* < .001). There were no significant differences in rates of maternal readmission or puerperal fever (Table 1).

**Table 1.** Maternal and Labor Characteristics

Characteristic	Individuals, No. (%) (N = 100 198)			
	Estimate of FGR		No estimate of FGR	
	Group 1-AGA (n = 5671)	Group 2-SGA (n = 3040)	Group 3-SGA (n = 8508)	Group 4-AGA (n = 82 979)
Maternal characteristic				
Maternal age, mean (SD)	28.3 (5.6)	27.5 (5.6)	27.9 (5.7)	28.8 (5.7)
Nulliparity	1429 (25.2)	1266 (41.6)	3275 (38.5)	19667 (23.7)
Gestational diabetes	151 (2.7)	71 (2.3)	163 (1.9)	2655 (3.2)
Hypertensive disorder	142 (2.5)	155 (5.1)	285 (3.3)	1420 (1.7)
Assisted reproductive technology	208 (3.7)	154 (5.1)	342 (4.0)	2281 (2.7)
Other maternal clinical condition	1044 (18.4)	597 (19.6)	1284 (15.1)	12249 (14.8)
Prior cesarean delivery	935 (16.5)	339 (11.2)	899 (10.6)	8927 (10.8)
Prior miscarriage	1612 (28.4)	755 (24.8)	2120 (24.9)	23645 (28.5)
Labor characteristic				
Spontaneous onset of labor	4343 (76.6)	1781 (58.6)	6955 (81.7)	72234 (87.1)
Induction of labor	649 (11.4)	969 (31.9)	1055 (12.4)	6964 (8.4)
Elective cesarean delivery	679 (12.0)	290 (9.5)	498 (5.9)	3782 (4.6)
Spontaneous delivery	4508 (79.5)	2243 (73.8)	6674 (78.4)	71845 (86.6)
Instrumental delivery	248 (4.4)	241 (7.9)	728 (8.6)	4546 (5.5)
Cesarean delivery	915 (16.1)	556 (18.3)	1106 (13.0)	6588 (7.9)
Placental complications	148 (2.6)	104 (3.4)	295 (3.5)	2358 (2.8)
Hemoglobin drop >3 g/dL	305 (5.4)	189 (6.2)	595 (7.0)	4906 (5.9)
Use of blood products	50 (0.9)	36 (1.2)	93 (1.1)	701 (0.8)
Puerperal fever	14 (0.2)	12 (0.4)	25 (0.3)	188 (0.2)
Maternal readmission	20 (0.4)	11 (0.4)	51 (0.6)	389 (0.5)

Abbreviations: AGA, appropriate for gestational age; FGR, fetal growth restriction; SGA, small for gestational age.

SI conversion factor: To convert hemoglobin to grams per liter, multiply by 10.0.

Multivariable logistic regression models comparing group 1-FP, group 2-TP, and group 3-FN with group 4-TN were fitted to estimate the independent association of antenatal fetal weight estimation with CD and ID rates (Table 2). Models were adjusted for maternal and neonatal covariates. False-positive FGR diagnosis (ie, group 1-FP) was independently associated with an increased CD rate, with an increase in risk of more than 70% (OR, 1.72; 95% CI, 1.56-1.88; *P* < .001).

**Table 2.** Multivariate Logistic Regression for CD and ID vs Group 4-TN

Outcome	Group^a^	OR (95% CI)
CD^b^	group 1-FP	1.72 (1.56-1.88)
	group 2-TP	1.06 (0.91-1.22)
	group 3-FN	0.78 (0.69-0.88)
ID^c^	group 1-FP	0.77 (0.67-0.88)
	group 2-TP	1.40 (1.16-1.68)
	group 3-FN	1.50 (1.32-1.71)

Abbreviations: AGA, appropriate for gestational age; CD, cesarean delivery; FN, false negative; FP, false positive; OR, odds ratio; SGA, small for gestational age; TN, true negative; TP, true positive.

^a^ Group 1-FP (ie, neonates with suspected fetal growth restriction who were AGA), group 2-TP (ie, neonates with suspected fetal growth restriction who were SGA), and group 3-FN (ie, neonates not suspected to have fetal growth restriction who were SGA) were compared with the reference group (ie, group 4-TN, neonates not suspected to have FGR who were AGA).

^b^ Risk for CD was adjusted for maternal age, nulliparity, prior cesarean delivery, prior miscarriage, maternal hypertensive disorder, use of assisted reproductive technology, any background maternal clinical condition, and neonatal birth weight.

^c^ Risk for ID was adjusted for maternal age, nulliparity, prior cesarean delivery, prior abortion, maternal hypertensive disorder, use of assisted reproductive technology, any background maternal clinical condition, and neonatal birth weight.

True-positive diagnosis of FGR (ie, group 2-TP) was not associated with risk of CD (OR, 1.06; 95% CI, 0.91-1.23; *P* = .49). A subanalysis of the data revealed that the increased CD rate in this group (18.3%) was associated with higher odds of nulliparity (OR, 5.47; 95% CI, 5.10-5.89; *P* < .001). When nulliparity was excluded from covariates, suspicion of FGR was associated with CD rate in group 2-TP (OR, 1.18; 95% CI, 1.02-1.37; *P* = .03). Lack of suspicion of FGR, despite SGA birth weight in group 3-FN, was associated with decreased CD risk (OR, 0.78; 95% CI, 0.70-0.88; *P* < .001).

For ID, FGR false-positive diagnosis (ie, group 1-FP) was associated with decreased risk of ID (OR, 0.77; 95% CI, 0.67-0.88; *P* < .001). For group 2-TP (ie, true-positive SGA), the model showed an association with increased ID risk (OR, 1.40; 95% CI, 1.16-1.68; *P* < .001). When SGA was not suspected (ie, group 3-FN), an association with increased ID risk was found (OR, 1.5; 95% CI, 1.32-1.71; *P* < .001).

### Neonatal Outcomes

Mean (SD) birth weight was 3165 (359) g for group 1-FP, 2496.3 (277) g for group 2-TP, 2630.94 (234) group 3-FN, and 3323.77 (158) for group 4-TN. Among 8711 neonates with suspected FGR, 34.9% were below the 10th percentile of birth weight according to national population-based growth curves, while 65.1% were AGA.^16^

Significantly different rates of Apgar score below 7 for neonates who were SGA compared with those who were AGA (ie, group 2-TP and group 3-FN vs group 1-FP and group 4-TN) were found at 1 minute (149 neonates [4.9%] and 384 neonates [4.5%] vs 124 neonates [2.2%] and 1595 neonates [1.9%]; *P* < .001) and 5 minutes (42 neonates [1.4%] and 143 neonates [1.7%] vs 22 neonates [0.4%] and 383 neonates [0.5%]; *P* < .001). Similarly, increased NICU admission rate was found for SGA groups (ie, group 2-TP and group 3-FN) compared with AGA groups (ie, group 1-FP and group 4-TN) (182 neonates [6.0%] and 328 neonates [3.9%] vs 51 neonates [0.9%] and 704 neonates [0.8%]; *P*  <.001), as were rates of prolonged NICU stay (ie, >72 hours; 151 neonates [5.0%] and 175 neonates [2.1%] vs 27 neonates [0.5%] and 321 neonates [0.4%]; *P* < .001) and prolonged neonatal hospitalization (564 neonates [18.5%] and 1010 neonates [11.9%] vs 592 neonates [10.4%] and 5139 neonates [6.2%]; *P* < .001) (Table 3).

**Table 3.** Neonatal Characteristics and Outcomes

Characteristic	Neonate, No. (%) (N = 100 198)				*P* value
	Estimate of FGR		No estimate of FGR		
	Group 1-AGA (n = 5671)	Group 2-SGA (n = 3040)	Group 3-SGA (n = 8508)	Group 4-AGA (n = 82 979)	
Mean birth weight, mean (SD), g	3165 (359)	2496.3 (277)	2630.94 (234.64)	3323.77 (158.66)	<.001
Apgar 1 min <7	124 (2.2)	149 (4.9)	384 (4.5)	1595 (1.9)	<.001
Apgar 5 min <7	22 (0.4)	42 (1.4)	143 (1.7)	383 (0.5)	<.001
NICU admission	51 (0.9)	182 (6.0)	328 (3.9)	704 (0.8)	<.001
NICU for 72 h	27 (0.5)	151 (5.0)	175 (2.1)	321 (0.4)	<.001
Prolonged neonatal hospitalization (>5 d)	592 (10.4)	564 (18.5)	1010 (11.9)	5139 (6.2)	<.001

Abbreviations: AGA, appropriate for gestational age; FGR, fetal growth restriction; NICU, neonatal intensive care unit; SGA, small for gestational age.

## Discussion

### Principal Findings

This cohort study found that neonates suspected of FGR had increased rates of obstetric intervention, including induction of labor and primary and overall CD, regardless of actual birth weight. In multivariate analysis, suspicion of FGR in neonates who were AGA was independently associated with an increase in risk for CD by more than 70%. However, in neonates who were SGA, there was no independent association of suspicion of FGR with increased CD, and the increased CD rate in this group was associated with maternal background characteristics, such as nulliparity. Regardless of suspicion of FGR, neonates who were SGA had decreased Apgar scores and increased NICU admission rates.

### Poor Antenatal Detection of Small Fetuses

More than 65% of neonates antenatally suspected for FGR were AGA birth weight as recorded after delivery. However, these neonates had significantly increased rates of obstetric intervention, including overall and primary CD rate and induction of labor. Our findings are in accordance with a previous publication^6^ that found poor antenatal detection of low birth weight and an increase in obstetric interventions. Antenatal detection of fetal weight is assessed via sonographic or clinical estimation, which depend on multiple factors that may be associated with the accuracy of the evaluation, including equipment, calculation formula, operator or clinician experience, and maternal body mass index.^18^ The reported percentage error is variable and considered to be approximately 10%. The agreement between sonographic and clinical assessment is high.^19^

Bricker et al^20^ found that routine late-pregnancy ultrasound in low-risk or unselected populations was not associated with benefits for the mother or baby but was associated with an increased CD rate. It may be inferred that poor detection of fetal weight is associated with a large part of the increase in CD in neonates who are AGA.

### Obstetrician Cognitive Bias and Risk of Cesarean Delivery

Our findings suggest the cardinal role of obstetrician cognitive bias in the process of obstetric decision-making. As found in the multivariate model in neonates who were AGA, suspicion of FGR was associated with an increase in risk of CD by more than 70%. This interesting finding may be explained by understanding social sciences concepts that interpret human behavior and decision-making under stress.

Confirmation bias may be an appropriate explanation for the association of antenatal assessment of FGR with mode of delivery, regardless of actual birth weight. Confirmation bias refers to the human tendency to interpret data in favor of prior beliefs to support a predetermined choice. Applying this bias to the course of events in the delivery suite, we may assume that the clinician has a baseline belief that a small fetus is compromised and has a high probability of experiencing adverse outcomes under contractions. It is well established that fetal heart rate is subjectively interpreted by experts and depends on their experience. We suggest that when analyzing fetal heart rate during labor, the obstetrician may be more prone to interpret heart rate abnormalities as severe decelerations in a fetus with suspected FGR. This outcome associated with confirmation bias may lead the obstetrician to advise for a prompt intervention via an instrumental or cesarean delivery. Similarly, we suggest that suspicion of FGR may be associated with clinical judgment of proper labor progress, hastening the decision to end labor with emergency CD. In other cases, mothers are not granted a trial of labor and undergo an elective CD as reflected by the increased rate of primary-elective CD in group 1-FP compared with group 4-TN (12.0% vs 4.6%).

Prospect theory was initially described by Kahneman and Tversky in 1979^21^ and deals mainly with monetary outcomes and financial decision-making under uncertainty. The basic principles regarding decisions with uncertain outcomes may be readily applied to obstetric management at admission to labor. According to prospect theory, human nature is risk-averse, meaning that individuals will take actions to avoid potential losses even at the sacrifice of more likely potential gains. Regarding clinical decision-making for mode and timing of delivery in a parturient with suspicion of FGR, the possible outcomes may be considered losses or gains in the obstetrician’s eyes. When a CD is medically justified, the outcome of not performing an urgent CD is considered a major loss given that the neonate may die or experience a severe disability. Alternatively, the possible gain of not performing a CD consists of avoiding immediate maternal morbidity and financial burden, along with economic and health consequences of repeat CD in the future. A risk-averse obstetrician may choose CD even when the probability of adverse neonatal outcome is small, because the loss looms larger than any potential gain that is not as salient as the loss.

Additionally, medical liability and the obstetrician’s sense of personal responsibility may be greater in the former scenario than the latter. When a prompt intervention is chosen and a healthy baby is born who is SGA or AGA, it may be difficult to question the intervention given that it seems that the main goal was achieved. Exceedingly high CD rates are not a priority in individual patient treatment, but rather the immediate obstetric outcome is the priority. These arguments may explain the predominance of CD when FGR is suspected among neonates who are AGA. Interestingly, in the multivariate model, the lack of suspicion of FGR was associated with a protective outcome, with a decrease in the risk of CD by 30%. Possibly, an antenatal estimation of adequate fetal weight may allow the clinician to be tolerable toward fetal heart rate changes and labor progress, allowing successful vaginal delivery.

Maternal characteristics, such as nulliparity, presence of hypertensive disorder, ART conception, and prior CD, were independent risk factors associated with CD and were found to be more prevalent in the population of neonates suspected of FGR. When adjusted for these factors, increased risk of CD in neonates with suspected FGR who were SGA was not associated with the suspicion but rather with other background maternal characteristics, mainly the increased rate of nulliparity. This may be associated with the expectation that multiparous mothers will have a shorter overall and second stage of labor duration,^22^ with a shortened period of anticipated fetal compromise during delivery, which may encourage clinicians to favor a trial of labor.

The rate of IDs among the false-positive group 1-FP was not higher than that of the true-negative group 4-TN (4.4% vs 5.5%). A possible explanation may be that ID is most often performed for nonreassuring fetal heart rate at the second stage of labor. Neonates who are AGA have less fetal distress than those who are SGA, who have fewer reserves to withstand labor stress and the interference of blood flow during contractions. This finding is further supported by the result that group 2-TP was comparable to group 3-FN in having an increased rate of ID, (7.9% and 8.6%, respectively).

### Adverse Maternal and Neonatal Outcomes Regardless of Antenatal Weight Assessment Among SGA Neonates

In our cohort, neonates who were SGA (ie, group 2-TP and group 3-FN) had increased rates of a low Apgar score, NICU admission, and prolonged hospitalization regardless of antenatal weight assessment. Concomitantly, mothers of neonates who were SGA had increased rates of postpartum placental complications (including manual lysis of retained placenta or placental fragments), postpartum hemorrhage, hemoglobin drop of more than 3 g/dL in the first 48 hours postpartum, and need for blood products. These findings are supported by previous reports of poor perinatal outcomes among neonates who were SGA, including increased mortality and cerebral palsy rates.^23,24,25,26^ The adverse outcomes may be explained by the natural pathophysiologic events commonly termed placental syndrome.^27,28^ The primary events of trophoblastic invasion and formation of proper placental blood vessels are crucial to the establishment of successful placental function that will further promote adequate fetal growth.^29,30,31^ Nulliparous mothers and those with ART conceptions are at risk of poor placental formation and increased vascular-based morbidity.^32,33^

In this cohort of neonates who were SGA, obstetric intervention was not associated with improved neonatal outcomes. This finding suggests that fetal insult may be chronic during pregnancy rather than acute intrapartum.

### Clinical and Research Implications

Constant vigilance and personal awareness of possible cognitive bias in obstetric decision-making is the first step toward limiting the association of these biases with maternal and neonatal outcomes among neonates who are AGA.

This study raises issues and hypotheses to be further investigated in future studies. First, future studies may need to use novel artificial intelligence techniques to attempt to increase the accuracy of sonographic weight estimation. Second, our findings suggest that novel algorithms for obstetric clinical decision-making should incorporate outcomes associated with confirmation bias and other cognitive biases into calculations of delivery management. Third, this study’s findings suggest that cost calculations should be performed to estimate the financial cost associated with unnecessary CD vs the possible expenses of neonatal disability or death.

### Limitations

This study has several limitations. First, the study’s retrospective nature prevents the determination of the causative effect of obstetrician’s cognitive bias and poor neonatal outcomes. Second, arterial pH values are not available for all neonates. Thus, pH data are not included in the study, although these data may contribute to the understanding of physiologic mechanisms underlying the poor neonatal outcomes. Third, umbilical cord Doppler values are not available for all neonates and thus not included in the study, although this information could improve the detection rate of fetuses at risk for poor neonatal outcomes. Fourth, placental pathology specimens are not available for all neonates. Thus, this information is not included in the study, although it may contribute to the understanding of the pathophysiology of increased hemorrhagic morbidity and third-stage placental complications among mothers of neonates who are SGA. At this point, we cannot provide long-term developmental outcomes of study group participants. Fifth, we did not discriminate clinical from sonographic weight estimation given that these data are recorded collectively in our patient records.

## Conclusions

We found that antenatal fetal weight estimation and diagnosis of FGR were independently associated with clinical decision-making, increased rate of induction of labor, and an increase in risk for CD by more than 70% in neonates who were AGA. Prospect theory and cognitive bias were applied to interpret our findings.
